# The impact of eating alone on food intake and everyday eating routines: A cross-sectional study of community-living 70- to 75-year-olds in Sweden

**DOI:** 10.1186/s12889-024-19560-0

**Published:** 2024-08-14

**Authors:** Amanda Björnwall, Patricia Eustachio Colombo, Ylva Mattsson Sydner, Nicklas Neuman

**Affiliations:** 1 Department of Food Studies, Nutrition and Dietetics, Uppsala University, Box 560, Uppsala, 751 22 Sweden; 2 Department of Biosciences and Nutrition, Karolinska Institutet, Stockholm, 171 77 Sweden; 3 Centre on Climate Change and Planetary Health, London School of Hygiene and Tropical Medicine, London, WC1E 7HT UK

**Keywords:** Eating alone, Older people, Food-related outcomes, Food intake, Everyday eating routines, Eating pattern

## Abstract

**Background:**

Eating is fundamental not only to survival and health, but also to how humans organise their social lives. Eating together with others is often seen as the healthy ideal, while eating alone is highlighted as a risk factor for negative health outcomes, especially among older adults. This paper, therefore, investigates the relationship between the frequency and subjective experience of eating alone and food-related outcomes among 70- to 75-year-olds in Sweden.

**Methods:**

A survey was distributed to a nationally representative random sample of 1500 community-living in Sweden, aged 70–75 years. Two different constructs of eating alone (objective and subjective) were measured, along with the following food-related outcomes: a food index, intake of food groups, consumption of ready-made meals, number of main meals per day, and body mass index (BMI).

**Results:**

In total, 695 respondents were included in the final sample. A quarter of the respondents were categorised as eating alone, of which a small proportion reported that doing so bothered them. There were no associations between eating alone and food index scores, BMI, or intake frequencies of fruits and berries, or fish and shellfish. However, people eating alone were less likely to report eating three meals per day (OR: 0.53, CI: 0.37–0.76, *p* = 0.006), less likely to report higher intake frequencies of vegetables and snacks, sugary foods, and sweet drinks (adjusted OR: 0.68, CI: 0.48–0.95, *p* = 0.023 resp. OR: 0.59, CI: 0.43–0.81, *p* = 0.001), and more likely to report higher intake frequencies of ready-made meals (adjusted OR: 3.71, CI: 2.02–6.84, *p* < 0.001) compared to those eating together with others. The subjective experience of eating alone did not have an impact on food-related outcomes.

**Conclusion:**

Eating alone or with others played a role in participants’ food intake, and seemed to influence aspects of the organisation of everyday eating routines rather than overall dietary healthiness or weight status. Our findings add to the previous body of research on commensality, eating alone, and health among the older population, providing insights into the development of future health policies and research.

**Supplementary Information:**

The online version contains supplementary material available at 10.1186/s12889-024-19560-0.

## Introduction

Eating is fundamental not only to survival and health, but also to how humans organise their social lives. As a consequence, eating in the company of others or in solitude can matter [1]. Some countries actively promote sharing meals as part of their public health advice [2–4], and the scientific data provide some support for this. The scientific concept used for the practice of eating together is commensality, a practice that is seen as a healthy ideal [1, 5, 6] with a variety of proposed benefits, both social [7] and nutritional [8, 9]. Studies directed at older people eating alone largely confirm this, demonstrating associations with several negative health- and food-related outcomes, which seem to be particularly prominent among this group [7, 10]. Apart from psychosocial aspects, such as an increased risk of depression [11] and cognitive decline [12], eating alone is also associated with lower food diversity [13, 14], lower intake of fruit and vegetables [15], lower caloric intake [16], meal skipping, and both under- and overweight [15]. Research highlighting the negative impacts of eating alone among older people generally covers a wide age interval, where individuals 60 years and above are included in the same study, thereby encapsulating a large variety of health statuses, abilities, and needs [12–16]. However, the links between eating alone and food-related outcomes may vary within the population group classified as older [10], and the needs of this group may also vary. Nevertheless, studies on eating alone and food-related outcomes that particularly target the earlier stages of retirement and old age are scarce [10].

In this paper, we address a potential issue that has been identified which concerns how eating alone is operationalised, that is when eating alone as an abstract concept is transformed into measurable observations. So far in research on commensality, ‘eating alone’ has been treated in a unidimensional and objective way, that is simply assessing whether or not a person eats alone (in solitude) or together with someone [10]. This approach has important empirical and theoretical drawbacks. Empirically, qualitative studies demonstrate that older people’s experiences of eating alone differ markedly. Some report how it makes them feel lonely, miss a partner, and lose interest in both cooking and eating [17, 18]. This can result in simplifications of everyday cooking and the reorganisation of mealtimes, sometimes described as leading to food of lower perceived quality (e.g., convenience foods) [19, 20]. For others, it is an unproblematic routine and natural part of the day, with ready-made meals and other convenience foods being considered positive [20–22]. The theoretical drawbacks are evidenced if we turn to the research on social relations, where the literature demonstrates the importance of distinguishing between being alone in an objective manner and the subjective feelings of loneliness [23]. These two separate constructs can have different health implications [24, 25], yet the commensality literature currently lacks such a distinction. Levels of loneliness (as a subjective experience) are relatively low in Sweden compared to other European countries [26–28]. At the same time, almost one third of Swedish older adults live in single households and it is the second most common living arrangement [29]. As such, this study is located in a national context in which many people live alone (objectively) yet a comparatively small group of people feel lonely. However, according to a Nordic study, most Swedes over the age of 60 years eat most of their meals with someone [30]. This seemed to be related to other aspects of the organisation of daily routines, such as the duration of meals, and if they are eaten in front of the TV, or sitting down at the kitchen table.

To summarise, we have theoretical reasons to anticipate that people can eat alone quite often without being bothered by it, an anticipation that is deduced in the literature on loneliness and social isolation yet unacknowledged in the commensality literature. The subjective experience of eating alone may, therefore, play a role in understanding the relationship between eating alone and previously identified food-related outcomes, such as the intake of different food groups, ready-made meals, weight status, and everyday eating routines. This is once again, similar to the way the subjective experience of loneliness is of importance for the effects of being alone. Against this backdrop, we aimed to investigate the relationship between eating alone (measured both objectively and subjectively) among 70- to 75-year old, community-living people in Sweden, and food-related outcomes (food index score, intake of food groups, consumption of ready-made meals, number of main meals per day, and body mass index [BMI]).

## Methods

This study was based on a cross-sectional, self-reported survey from a random and nationally representative sample of 70- to 75-year-olds in Sweden. In total, 1500 people were invited to participate and given the opportunity to respond to the survey either digitally or with pen and paper.

### Data collection and Respondents

The survey was distributed nationally by post to randomly selected individuals retrieved from the Swedish state personal address register. An invitation letter with information about the study, a QR-code, and a link to reach the web-based survey was sent out in November 2021. Two reminders (December 2021 and January 2022) were sent to those who had not replied and not actively declined participation. The second and final reminder included a paper copy of the survey and a stamped self-addressed envelope. Data were collected and managed in REDCap - a secure, web-based software platform, designed to support data capture for research studies [31]. The COVID-19 pandemic was ongoing during the data collection phase, although, by that time, restrictions had been lifted and the majority of those in Sweden over 70 years of age had been vaccinated. However, around the time of the second reminder, new restrictions on physical contacts were temporarily in place due to a new disease wave [32].

The age category of 70–75 years was chosen for mainly two reasons. First, a majority of older people in Sweden (and Europe) are healthy, active, and independent [33, 34]. In line with this, retirement ages are increasing, and people are working beyond retirement age; the standard Swedish cut-off of 65 years or older therefore seemed too young for our purposes. Furthermore, this study targets an early phase of retirement, a time of finding or having found new routines after working life. It is, therefore, of interest to investigate whether the possible disadvantages of eating alone are evident at this rather early stage of later life. Even though there are people older than 75 years who are still working, an older target group did not seem relevant for this purpose.

**Fig. 1:**
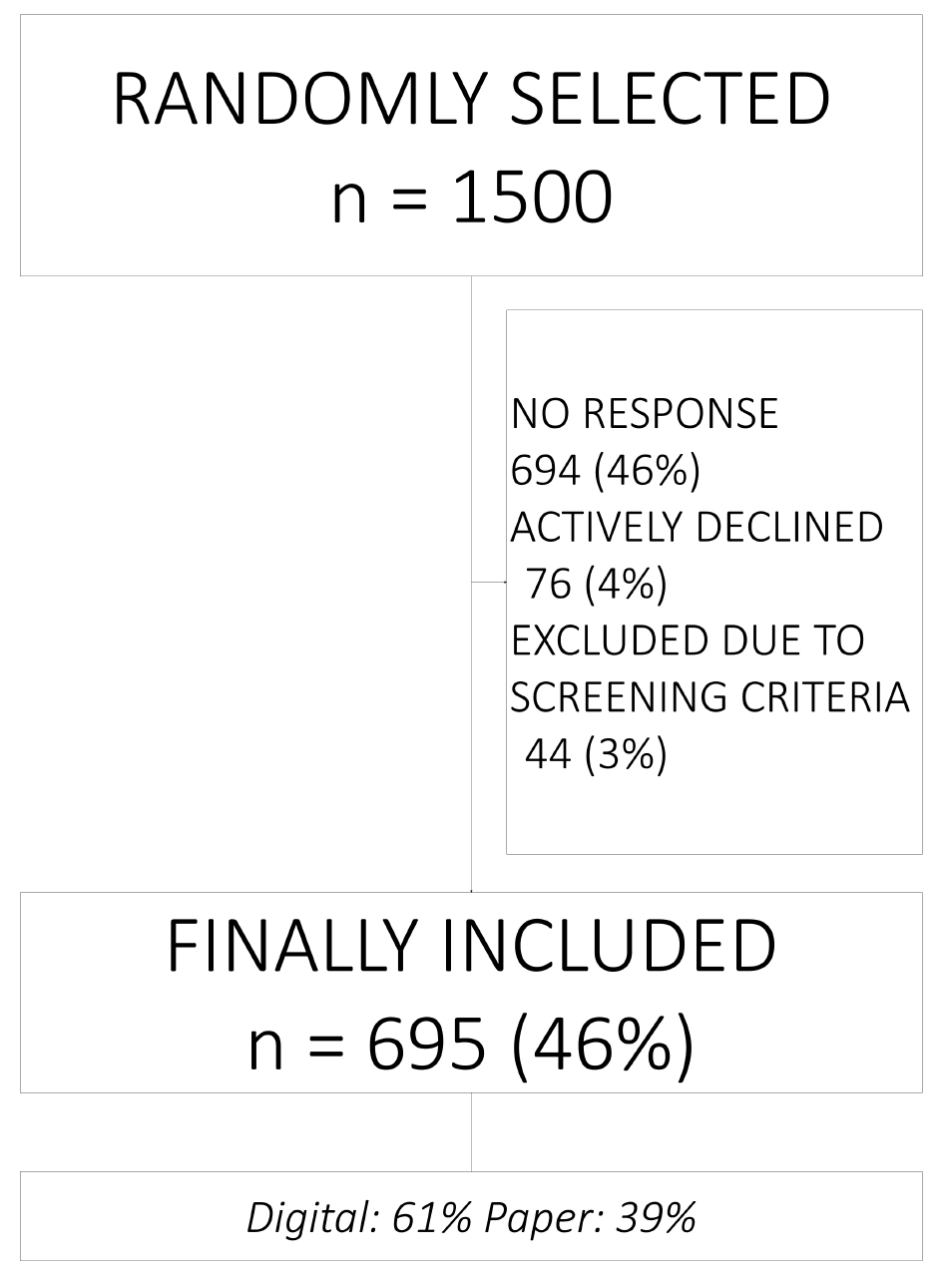
Flowchart of study participants.

Second, including a restricted age category was decided upon based on power calculations that showed that a larger sample size would have been needed to be able to perform age-specific analyses. A power calculation with 95 per cent confidence interval and unknown proportion of people eating alone in the particular age category resulted in a preferred sample size of at least 385 respondents [35]. Considering the decrease in response rates to national surveys over the past years [36], the survey group invited to participate was more than three times larger than needed (*n* = 1500). Exclusion criteria were individuals diagnosed with or under medical investigation for dementia and those living in a long-term care facility; these were determined through self-reported screening questions. The survey was only distributed in Swedish and informed consent was needed for responses to be included. This resulted in a final study sample of 695 respondents (Fig. 1).

### Survey development

The survey was developed in an iterative process. The majority of questions included were re-used (either literally or with minor modifications) from large national studies [37–39]. Senior researchers with experiences of research in older peoples’ health and nutrition reviewed the survey at an initial stage and provided feedback on its content and questions. The survey was then further developed by holding cognitive interviews [40] with five individuals belonging to the target population, resulting in changed linguistic formulations and descriptions of questions. The questions regarding eating alone or together, that were created from scratch for this survey, were tested in a bachelor thesis in order to evaluate the data collection process, such as subjects’ willingness to participate, data entry, and feedback from respondents (provided as additional free-text responses at the end of the survey). Finally, a pilot study of the survey in its entirety, including people from the target population (*n* = 177), was performed. The pilot did not result in any changes to the survey, but it did provide us with a relevant dataset for testing the statistical models. A version of the survey translated into English can be accessed in Supplementary File A. This is attached for reasons of transparency, so that readers can see the questions asked. However, it has not been tested or validated for use in an English-speaking population.

## Data and variables

### Eating alone

The frequency of eating alone or together with someone was assessed using the question “How often do you eat together with someone?”, for which the responses were “Daily”, “Several days per week”, “One or two days per week”, “One or two days per month”, “Less often or never”. The responses to these questions were then transformed into a binary variable, with those reporting eating together with someone daily categorised as *eating together* and those reporting eating together with someone less often than that categorised as *eating alone*. The reason for this was that eating together with someone several days per week or less often means that a substantial proportion of meals are eaten alone.

The subjective experience of eating alone was assessed by asking “When you eat alone, does this ever bother you?”, for which the responses were “Always”, “Often”, “Rarely”, “Never”, or “I never eat alone”. Here too, a binary variable was constructed. Those responding “Rarely” or “Never” were categorised as *not bothered*. Those who reported that they never ate alone were also categorised as *not bothered*, since they could not be bothered by something they never did (i.e., they were not exposed to the phenomenon that could possibly influence the dependent variable). The remaining responses were categorised as *bothered*. Eating alone was not defined or specified in a particular way, e.g., as being alone in the room, at the table, or being the only one eating. Instead, it was up to the respondents to interpret what eating alone meant to them.

### Food-related outcomes

The food-related outcomes consisted of a food index, intake of specific food groups, consumption of ready-made meals, number of main meals per day, and BMI. The food index, developed by the Swedish Food Agency and the Swedish National Board of Health and Welfare, intends to capture food intake relevant from a public health perspective with questions that are easy to answer [41]. The index was constructed using a robust process for the purpose of finding accurate indicators of a diet more or less concordant with the Swedish Food Based Dietary Guidelines (FBDGs), which are consistent with the WHO recommendations [42]. The food index contains questions about the intake frequencies of four food groups (vegetables, fruits and berries, fish and shellfish, and snacks, sugary foods, and sweet drinks). Every question has four response categories which provide points from 0 to 3 (higher points for higher frequency of consumption, except for snacks, sugary foods, and sweet drinks which is scored in the opposite direction), adding up to a total of 12 points. For vegetables, fruits and berries, and snacks, sugary foods, and sweet drinks the response categories are “Two or more times per day”, “Once per day”, “A few times per week”, or “Once per week or less often”. The response categories for fish and shellfish are “Three or more times per week”, “Twice per week”, “Once per week”, or “A few times per month or more rarely”. So, for example, if a respondent reported consuming fruits and berries two or more times per day, they would receive 3 points for their response. A higher score indicates a diet better aligned with Swedish FBDGs. Respondents needed to have reported their frequency of consumption for the four food groups included in the food index to be able to calculate a score. The food index (sum score) was categorised into three groups based on the criteria set by the Swedish National Board of Health and Welfare: (1) *not eating according to recommendations* (score: 0–4), (2) *somewhere in-between* (score: 5–8), and; (3)* eating approximately according to recommendations* (score: 9–12). Intake frequencies of the four food groups were also analysed separately, using the response categories stated above, but not the scoring system.

Intakes of ready-made meals and main meals were assessed by asking “How often do you eat the following: Ready-made meals, Breakfast, Lunch, Dinner?”, for which the responses were “Daily”, “Several days per week”, “One or two days per week”, “One or two days per month”, “Less often or never”. The responses for ready-made meals were categorised as *often* (“Daily”, “Several days per week”), *sometimes* (“One or two days per week”), and *rarely or never* (“One or two days per month”, “Less often or never”). To generate a total number of main meals per day, respondents needed to have responded to each of the three questions regarding main meals. Eating one main meal per day gave one point, making it possible to have a total of 0–3 main meals per day. Eating three main meals per day is the conventional meal pattern in Nordic (and other European) countries [30, 43], which the majority of respondents in this study also did. This variable was therefore categorised as binary, divided into eating three [3], or two or fewer meals per day (≤ 2). We treated these two variables as factors related to the organisation of everyday eating routines, both of which influence food and eating activities [44]. Lastly, BMI was estimated using information on self-reported height in centimetres and self-reported weight in kilograms. Respondents were not given specific instructions on how to measure their height or weight.

### Other variables

Year of birth, sex (female or male), living situation (cohabiting or living alone), marital status (not married, married/cohabiting, divorced, or widowed), and country of birth (Sweden, Nordic, European, or other) were included in the survey. Educational level was categorised into primary (< 10 years), secondary (10–12 years) and post-secondary (≥ 13 years) education. Respondents were also asked to rate their general health status on a five-point scale, ranging from “Very good” to “Very poor”.

### Statistical analysis

For all statistical computations, the software R version 4.3.0 was used [45], with embedded functions as well as the R packages ‘tidyverse’ [46], ‘ggplot’ [47], ‘jtools’ [48], ‘car’ [49], ‘ordinal’ [50], and ‘rcompanion’ [51]. Descriptive statistics were computed with proportions, means, and standard deviation (if continuous variable). Simple and multiple logistic regressions were used to examine associations between the frequency of eating alone (eating alone vs. eating together) and number of main meals per day (binary variable), and simple and multiple ordinal regressions were used to examine the associations between the frequency of eating alone and food index scores, intake of food groups, consumption of ready-made meals (ordinal variables). The association between the frequency of eating alone and BMI (continuous variable) was assessed with simple and multiple linear regression.

In the multiple regression models, we included the subjective experience of eating alone (bothered vs. not bothered) as an independent variable to explore its independent effect on outcomes. Including this variable also meant that we were able to control that the variation explained by the frequency of eating alone (i.e., objective measure) was independent. Adjustments were initially made for the following covariates: sex, living situation, and educational level. Multicollinearity between independent variables was considered by computing variance inflation factors (VIF). Multicollinearity (VIF > 2) was found between the main predictor eating alone or together, and the independent variable living situation. Living situation was, therefore, not included as an independent variable in the adjusted models and the models were only adjusted by sex and educational level. The reason for choosing education as the measure of socioeconomic status is that educational level has become an increasingly important social stratifier in post-industrial European welfare states, and is insensitive to reverse causation (i.e., your completed education at timepoint 1 cannot be affected by your health at timepoint 2) [52]. The significance level for all statistical analyses was set at p-value < 0.05. Model outputs were presented with p-values and 95 per cent confidence intervals (CI).

### Ethical considerations

Approval for this study was sought from and approved by the Swedish Ethical Review Authority (Dnr 2021 − 01988). Informed consent was obtained from all respondents.

## Results

### Study population

A description of the study population is presented in Table 1. There was a fairly equal distribution of male and female respondents in the study population, however with more women eating alone. More than two thirds of the study population had secondary or post-secondary education (≥ 10 years), and over 90 per cent of the respondents were born in Sweden. Three quarters rated their general health status as good or very good, one quarter as moderate, and very few rated their health status as poor or very poor. These characteristics were equally distributed among the two groups (eating alone and eating together). Age was also equally distributed in the total sample, as well as between the two groups, and was further treated as one age category. The majority of the respondents, about three quarters, were cohabiting and one quarter were living alone. This coincided with eating alone or together, meaning that most people who were cohabiting were eating together and most people living alone were eating alone.

**Table 1:** Characteristics of the study population (*n* = 695)

**Characteristics**	All		Eating together		Eating alone	
	*N* = 695	%	*N* = 503	%	*N* = 192	%
**Sex**						
Female	366	53	242	48	124	65
Male	319	46	253	50	66	34
N/A	10	1	8	2	2	1
**Education (y)**						
Primary (< 10)	188	27	139	28	49	25
Secondary (10-12)	194	28	135	27	59	31
Post-secondary (> 13)	301	43	222	44	79	41
N/A	12	2	7	1	5	3
**Health status**						
Very good	154	22	111	22	43	22
Good	369	53	277	55	92	48
Moderate	149	22	100	20	49	26
Poor	18	3	11	2	7	4
Very poor	2	0	2	0.5	-	
N/A	3	0	2	0.5	1	0
**Age**						
70	108	15	76	15	32	17
71	123	18	91	18	32	17
72	102	15	75	15	27	14
73	124	18	92	18	32	17
74	101	14	78	16	23	12
75	116	17	82	16	34	17
N/A	21	3	9	2	12	6
**Living situation**						
Living alone	166	24	4	1	162	85
Cohabiting	527	76	498	99	29	15
N/A	2	0	1	0	1	0
**Marital status**						
Not married	79	12	21	4	58	30
Married/Cohabiting	502	72	476	95	26	14
Divorced	63	9	1	0	62	32
Widowed	48	7	4	1	44	23
N/A	3	0	1	0	2	1
**Country of birth**						
Sweden	633	91	464	92	169	88
Nordic	29	4	17	4	12	6
European	20	3	11	2	9	5
Other	12	2	10	2	2	1
N/A	1	0	1	0	-	

### Objective and subjective constructs of eating alone

The majority of the respondents reported eating together with someone daily (*n* = 503, 72%) and almost 16 per cent (*n* = 109) of the total sample reported that they never ate alone. Over one quarter of the sample reported eating alone most of the time (*n* = 192, 28%), and four per cent (*n* = 30) of the total sample reported eating together with someone less often than once per month. Of those eating alone, the majority rarely or never felt bothered by it, while a small proportion often or always did. Further details of the combination of the objective and subjective constructs of eating alone are presented in Table 2.

**Table 2:** The combination of the objective and subjective constructs of eating alone, presented with numbers, *n* = 695, and column %. Both constructs were coded into binary variables for regression analysis. Eating together ‘daily’ was categorised as eating together, while ‘several days per week’ or more rarely were categorised as eating alone. The experience of eating alone was divided into bothered (‘often/always bothered’) or not bothered (‘rarely/never bothered/never eating alone’)

n (%)	Frequency of eating together					
	Daily	Several days a week	One or a couple of days a week	One or a couple of days a month	More rarely or never	
**Experience of eating alone**						
Often/Always bothered	12 (2)	1 (3)	3 (5)	8 (11)	1 (3)	
Rarely/Never bothered	382 (76)	29 (97)	54 (95)	67 (89)	29 (97)	
Never eating alone	109 (22)	N/A	N/A	N/A	N/A	
n (%)	503 (100)	30 (100)	57 (100)	75 (100)	30 (100)	

### Eating alone and food intake

Table 3 shows odds ratios, 95 per cent confidence intervals, and p-values from adjusted ordinal regression analyses of food index scores, food groups (separated), and intake of ready-made meals. The unadjusted ordinal regression model revealed no association between food index scores and eating alone or together (*p* = 0.288, data not shown). The adjusted model confirmed that there was no statistically significant difference in food index categorisation between those eating alone vs. together (OR: 1.03, CI: 0.72–1.47, *p* = 0.870). The distribution of the food index scores for the two groups (eating alone vs. eating together) is presented in Supplementary Fig. 1.

No differences between the intake frequencies of fruits and berries (OR: 0.92, CI: 0.67–1.27, *p* = 0.608) or fish and shellfish (OR: 0.79, CI: 0.57–1.10, *p* = 0.160) were found between those eating alone and those eating together. However, there was a significant difference showing that respondents eating alone were less likely to report higher intake frequencies of vegetables (OR: 0.68, CI: 0.48–0.95, *p* = 0.023) and snacks, sugary foods, and sweet drinks (OR: 0.59, CI: 0.43–0.81, *p* = 0.001). Respondents eating alone were more likely to report a more frequent intake of ready-made meals (OR: 3.71, CI: 2.02–6.84, *p* < 0.001).

**Table 3:** Associations between eating alone and food-related outcomes, presented with n (%) or, when applicable, mean [sd], odds ratios or coefficient, 95% confidence intervals and p-values

	All n (%) / mean [sd]	Eating together	Eating alone	OR (95%CI)	*P*
**Food-related outcomes**					
Food index*	688 (100)	501 (100)	187 (100)	1.03 (0.72–1.47)	0.870
0–4	68 (10)	48 (10)	20 (11)		
5–8	419 (61)	315 (63)	104 (55)		
9–12	201 (29)	138 (27)	63 (34)		
Food groups*					
*Fruits and berries*	693	503	190	0.92 (0.67–1.27)	0.608
Two or more times per day	201 (29)	142 (28)	59 (31)		
Once per day	305 (44)	227 (45)	78 (41)		
A few times per week	133 (19)	99 (20)	34 (18)		
Once per week or less often	54 (8)	35 (7)	19 (10)		
*Vegetables*	694	503	191	0.68 (0.48–0.95)	0.023
Two or more times per day	143 (21)	103 (20)	40 (21)		
Once per day	377 (54)	288 (57)	89 (46)		
A few times per week	151 (22)	98 (19)	53 (28)		
Once per week or less often	23 (3)	14 (3)	9 (5)		
*Fish and shellfish*	693	502	191	0.79 (0.57–1.10)	0.160
Three or more times per week	91 (13)	64 (13)	27 (14)		
Twice per week	245 (35)	183 (36)	62 (33)		
Once per week	281(41)	210 (42)	71 (37)		
A few times per month or less often	76 (11)	45 (9)	31 (16)		
*Snacks, sugary foods, and sweet drinks*	691	502	189	0.59 (0.43–0.81)	0.001
Two or more times per day	43 (6)	31 (6)	12 (6)		
Once per day	200 (29)	166 (33)	34 (18)		
A few times per week	229 (33)	164 (33)	65 (34)		
Once per week or less often	219 (32)	141 (28)	78 (41)		
Ready-made meals*	652	467	185	3.71 (2.02–6.84)	< 0.001
Often	7 (1)	1 (0)	6 (3)		
Sometimes	44 (7)	24 (5)	20 (11)		
Rarely/Never	601 (92)	442 (95)	159 (86)		
Main meals per day**	655 (100)	477 (100)	178 (100)	0.53 (0.37–0.76)	0.006
Two or fewer	297 (45)	197 (41)	100 (56)		
Three	358 (55)	280 (59)	78 (44)		
				Coefficient	
BMI***	25.98 [4.27]	25.88 [4.08]	26.23 [4.72]	0.55 (-0.19-1.29)	0.14

* Multiple ordinal regression model with food index, food groups, or ready-made meals as dependent variables; eating alone or together (objective), experience of eating alone (subjective), sex, and education as independent variables

** Multiple logistic regression model with main meals per day as dependent variable; eating alone or together (objective), experience of eating alone (subjective), sex, and education as independent variables

***Multiple linear regression model with BMI as dependent variable; eating alone or together (objective), experience of eating alone (subjective), sex, and education as independent variables

### Eating alone and the number of main meals per day

Table 3 shows the number and proportion of respondents eating three vs. two or fewer main meals per day in the two groups (eating alone vs. eating together), along with odds ratio, 95 per cent confidence interval, and p-value from the adjusted logistic regression model. The majority of respondents eating alone reported eating two or fewer meals per day (56%). In comparison, a majority of those eating together with someone were eating three meals per day (59%). The unadjusted model showed a statistically significant association between number of main meals per day and eating alone or together (*p* < 0.001, data not shown). The adjusted model confirmed this, showing that respondents eating alone were less likely to report eating three meals per day compared to those eating together with someone (OR: 0.53, CI: 0.37–0.76, *p* = 0.006).

### Eating alone and BMI

The distribution of BMIs among the two groups (eating alone vs. eating together) is presented in Supplementary Fig. 2. Table 3 shows the mean and standard deviation of BMI, coefficient, and p-value from the adjusted linear regression model. The simple linear regression model showed no significant association between BMI and eating alone or together (*p* = 0.27, data not shown). The adjusted model confirmed that there was no significant difference in BMI among respondents eating alone or together (B: 0.55, CI: -0.19-1.29, *p* = 0.14).

### Subjective experience of eating alone and food-related outcomes

There were no associations found between the subjective experience of eating alone (being bothered vs. not being bothered) and any of the food-related outcomes, except for the intake frequency of fish and shellfish. Here, those bothered by eating alone were less likely to report higher intake frequencies of fish and shellfish (OR: 0.36, CI: 0.16–0.80, *p* = 0.012, data not shown).

## Discussion

This study investigated the subjective and objective constructs of eating alone, and its relationship with food-related outcomes. Our results show that over a quarter of the sample of 70- to 75-year-olds living in Sweden are eating alone most of the time, and a small group of individuals reported being bothered by it. There were no differences identified in food index scores, intake frequencies of fruits and berries, fish and shellfish, or BMI between those categorised as eating alone or eating together with someone. However, participants eating alone reported a less frequent intake of vegetables and snacks, sugary foods, and sweet drinks, a more frequent intake of ready-made meals, and consuming fewer main meals per day than those eating together with someone. Additionally, the subjective experience of eating alone did not influence food-related outcomes, except for intake frequency of fish and shellfish, which was significantly lower among those being bothered by eating alone. Thus, it appears that, in this sample, eating alone is a better predictor of aspects related to the organisation of everyday eating routines rather than the overall healthiness of diet or the participants’ weight status.

Previous research has shown multiple negative food-related effects of eating alone among older adults [13–16], and there may be several reasons for why our study, in contrast, found no associations between eating alone and most food-related outcomes. Firstly, the study sample consists of independent and rather healthy older individuals in an early phase of retirement. It could be that the negative effects of eating alone become more prevalent and more severe in higher age brackets, since previous studies with wider age intervals (60 years and above) have identified such effects [13–16]. Secondly, the results may also be explained by cultural factors. Cultural individualism has been identified to modify the relationship between loneliness and health outcomes [53], something that may also be applied to the phenomenon of eating alone. Sweden is a country where the majority culture is considered individualistic [54] and single households are common in all adult age groups [29]. Our data, and that of others’, [30] show that commensality is the most common form of eating in Sweden, and qualitative studies of several age groups indeed suggest that commensality remains idealised, appreciated, and desired [55–58]. However, as we have shown in a qualitative interview study of Swedes aged 70 years and older, experiences and perceptions of eating alone differ widely [20]. Thus, it could simply be the case that for many people in Sweden, eating in solitude is not such a big deal, even if company would be preferred.

One aspect in which our findings agree with previous studies is the association between eating alone and the pattern of consuming fewer main meals per day or meal skipping [15], less frequent intake of vegetables [13, 15], and with descriptions of more frequent intake of ready-made meals [20]. In our study, those eating alone also reported less frequent intake of snacks, sugary foods, and sweet drinks. Even though these food-related outcomes can be related to health, we cannot draw any clear health-related conclusions from our data since they are only based on frequencies. Nevertheless, we do not reject the possibility that these outcomes could be problematic for older people at risk of malnutrition or other health-related issues where intake frequency is relevant. The motivation for consuming ready-made meals may vary for older adults, with factors such as cooking skills (especially among widowed men) and physical limitations affecting the preparation of food potentially impacting the demand for such meals [19, 22, 59]. The pattern of consuming fewer main meals and more ready-made meals seems therefore, first and foremost, to reflect the daily organisation of eating routines, for example indicating a simplification of everyday eating and less time spent cooking. As such, our results are more in line with sociological studies on how social factors are related to the eating routines of everyday life [44, 60] than studies on food intake and meal frequency and health. One particular hypothesis derived from our findings could be that eating alone influences the organisation of everyday eating, for example through eating fewer main meals and more ready-made meals, and that this triggers health implications later in life. Future research will have to test this hypothesis.

It is well documented that people tend to eat more in the company of others, referred to as the social facilitation of eating [61], while eating alone can have the opposite effect [16]. Hypothetically, weight loss could be an effect of eating alone since it is associated with meal skipping [15] and, in the present study, with eating fewer main meals. However, it is currently unclear whether eating alone and weight status are related. Previous research from Japan indicates an increased risk of self-perceived weight loss [62], and an increased risk of both under- and overweight among men eating alone, although not women [15]. As such, our findings add to the uncertainty of these mixed results, with no associations identified between eating alone and BMI.

The subjective experience of eating alone (bothered vs. not bothered) did not have an independent effect on food-related outcomes. This could be explained by the fact that few (only four per cent) in our sample report being bothered by eating alone. Since power calculations were based on the objective measure of eating alone, a larger sample size might have been able to detect a significant effect of the subjective experience of eating alone on food-related outcomes. Consideration of the subjective experience of eating alone in future research is likely to be important for capturing the full extent of links between eating alone and food-related outcomes.

### Strengths and limitations

This study is, to our knowledge, the first to consider both the objective and subjective constructs of eating alone. This was conducted through a rigorous and systematic procedure of questionnaire development, where questions were carefully chosen from, or based on, established surveys. To increase validity and reliability, the survey was further evaluated using pre-testing of eating-alone questions, a pilot study of its entirety, and cognitive interviews. Data collection was meticulously planned and structured to enable a high response rate, and we consider a response rate of almost half of the sample to be decent, albeit lower than optimal. However, a low response rate among lower educational groups, those with lower self-perceived health status, and a very small representation of non-European immigrants complicates the representativeness and thus generalisation of our findings. There is also the possibility that our sample is biased by the overrepresentation of individuals particularly interested in food and meals, since information about the topic of the study was given prior to participation.

The survey was distributed during the COVID-19 pandemic, which may have further impacted the generalisation of our results. Additionally, restrictions on physical contacts changed during the later data collection phase, meaning that the frequency of eating together or alone might have temporarily changed for some participants. Since people aged 70 years or older had been recommended to stay at home and minimise physical contacts with others during the pandemic, patterns of meal company could have been different compared to normal (non-pandemic) circumstances. This situation could also have affected food choice and food intake, for example due to changes in food shopping and access to food. As demonstrated by a recently published scoping review, such effects on older adults in different countries differ markedly [63]. For example, whereas an increased intake of several food groups (e.g. vegetables, sugary foods and snacks) during the pandemic was reported in some studies, others identified higher levels of food insecurity and hence a reduction in food intake.

In this study, BMI is used as an indicator of weight status. BMI has been criticised for not being a fully appropriate metric for evaluating the weight status of a given older patient, yet it has good predictive value at a population level [64] as long as the same cut-offs as for younger adults are not considered appropriate to apply [65]. Moreover, this study is based on self-reported data, which can be particularly problematic regarding height, weight, and food intake [66, 67]. No instructions were given for how height and weight should be measured, which adds another layer of uncertainty to the data. One possibility for handling self-reported BMI data is to use standardised factors for corrections [66, 68]. Such factors are of great value for improving precision in prevalence numbers based on self-reports. However, prevalence numbers as such were not the aim of this study, but rather associations with eating alone (or not). Food intake was also based on frequencies of food groups, which means that we cannot comment on absolute quantities or fine-grained dietary details (e.g., exactly which fruits and vegetables or which types of fish or shellfish). Further research is, therefore, warranted in order to test differentiated constructs of eating alone in relation to the food-related outcomes that are of particular clinical or public health interest.

## Conclusion

The present study consideres the differentiation between objective and subjective constructs when studying eating alone and its relationship with food-related outcomes among 70- to 75-year-old people in Sweden. In this sample of independent healthy individuals in an early phase of retirement, over a quarter of the respondents were eating alone, but few were bothered by this. There were no associations between the frequency of eating alone and level of healthiness of diet (food index scores), BMI, or intake frequency of fruits and berries, or fish and shellfish. However, people eating alone reported eating vegetables and snacks, sugary foods, and sweet drinks less often, consuming ready-made meals more often, and eating fewer main meals per day than those eating together. Eating alone, therefore, first and foremost seemed to be a predictor of outcomes related to the organisation of everyday eating routines, and indicating a simplification of cooking and eating. The subjective experience of eating alone had largely no impact on food-related outcomes. However, individuals bothered by eating alone are likely to be statistically underrepresented in our sample, which hinders our possibilities to draw conclusions in this regard. Despite the uncertainties raised, our findings add to the previous body of research on commensality, eating alone, and health among the older population, and provide insights into the development of future health policies and research.

### Electronic supplementary material

Below is the link to the electronic supplementary material.

Supplementary Material 1

Supplementary Material 2

## Data Availability

The datasets used and/or analysed during the current study are available from the corresponding author on reasonable request.
