# Supplementary material for: The impact of eating alone on food intake and everyday eating routines: A cross-sectional study of community-living 70- to 75-year-olds in Sweden
Source: BMC Public Health. 2024 Aug 14;24:2214. doi: 10.1186/s12889-024-19560-0 (PMC11323640; doi:10.1186/s12889-024-19560-0)
Supplement: Supplementary file 1 — Supplementary Material 1 [file 12889_2024_19560_MOESM1_ESM.pdf]

Supplementary file A

Survey study about meals and meal company

Introduction

Personal code: __ __ __ __

A) Informed consent is required to participate in the study:

- ☐ Yes, I consent to participate in the study *Eating alone or together with others among individuals aged 70 years and older*. I have received written information about the study, including contact information for researchers, and have had the opportunity to ask questions.
- ☐ No, I do not wish to participate in the study.

B) Have you been diagnosed with, or are under investigation for dementia?

- ☐ No
- ☐ Yes

C) What is your current living arrangement?

- ☐ Independent housing (e.g., apartment, house etc.)
- ☐ Senior community or sheltered housing (e.g., 55+, assisted living, etc.)
- ☐ Special housing or care facility (e.g., nursing home, care home, group home, etc.)

About me

1. Do you live alone?
   - ☐ No
   - ☐ Yes

2. If no, with whom do you currently reside? Please select all that apply.
   - o Spouse or partner
   - o Children or children-in-law
   - o Siblings
   - o Other

3. What is your current marital status?
   - ☐ Not married
   - ☐ Married/Cohabiting
   - ☐ Divorced/Former cohabitant
   - ☐ Widow/Widower

4. If Widow/Widower/Divorced, in which year did you become so?

   ______________ (YYYY)

5. Year of birth:

   ☐ 1951 ☐ 1950 ☐ 1949 ☐ 1948 ☐ 1947 ☐ 1946

6. In which country or region of the world were you born?
   - ☐ Sweden
   - ☐ Other Nordic country
   - ☐ Other European country
   - ☐ Other country

7. If you were born outside of Sweden, how many years have you lived in Sweden in total?

   ____

8. Sex:
   - ☐ Woman
   - ☐ Man
   - ☐ Don’t want to answer

9. Weight (kg): ______________

10. Height (cm): ______________

Health

11. How would you rate your overall health?
    - ☐ Very good
    - ☐ Good
    - ☐ Moderate/Fair
    - ☐ Poor
    - ☐ Very poor

12. Do you currently experience any chronic illnesses, injuries from accidents, limitations in function, or other long-standing health conditions?
    - ☐ No
    - ☐ Yes

13. If yes, do these discomforts result in reduced work capacity or hinder you in your other daily activities?
    - ☐ No, not at all
    - ☐ Yes, to some extent
    - ☐ Yes, to a high extent

Meal habits

The following questions pertain to how things usually are **on average, over time**.

14. How often do you eat vegetables and/or root vegetables?
    - ☐ Two or more times per day
    - ☐ Once per day
    - ☐ A few times per week
    - ☐ Once per week or less often

15. How often do you eat fruits and/or berries?
    - ☐ Two or more times per day
    - ☐ Once per day
    - ☐ A few times per week
    - ☐ Once per week or less often

16. How often do you eat fish or shellfish as a main course, in salad, or as topping?
    - ☐ Three or more times per week
    - ☐ Twice per week
    - ☐ Once per week
    - ☐ A few times per month or less often

Meal habits (continuing)

The following questions pertain to how things usually are **on average, over time**.

17. How often do you eat pastries, chocolate/candy, crisps, or soft drink/soda?
    - ☐ Two or more times per day
    - ☐ Once per day
    - ☐ A few times per week
    - ☐ Once per week or less often

18. How often do you typically eat the following?
    Please select the option that best applies to your situation and **mark one cross per row.**

	Daily	Several times per week	One or two times per week	One or two times per month	Less often
a) Breakfast	☐	☐	☐	☐	☐
b) Lunch	☐	☐	☐	☐	☐
c) Dinner	☐	☐	☐	☐	☐
d) Snack or in-between meal	☐	☐	☐	☐	☐
e) Meals outside home	☐	☐	☐	☐	☐
f) Ready-made meals	☐	☐	☐	☐	☐

Meal company

The following questions are about **the past three months**.

19. How often do you typically eat together with someone?
    - ☐ Daily
    - ☐ Several days per week
    - ☐ One or two days per week
    - ☐ One or two days per month
    - ☐ Less often or never

20. When you eat together with others, who do you typically eat with?
    - ☐ Spouse or partner
    - ☐ Children or children-in-law
    - ☐ Grandchildren
    - ☐ Friends
    - ☐ Other
    - ☐ I never eat with others

21. When you eat together with others, does this ever bother you?
    - ☐ Always
    - ☐ Often
    - ☐ Rarely
    - ☐ Never
    - ☐ I never eat with others

22. When you eat alone, does this ever bother you?
    - ☐ Always
    - ☐ Often
    - ☐ Rarely
    - ☐ Never
    - ☐ I never eat alone

23. If you would like company during meals, how often do you have the opportunity to do so?
    - ☐ Always
    - ☐ Often
    - ☐ Rarely
    - ☐ Never
    - ☐ Don't know/Prefer not to answer

Social relations and activities

The following questions are about **the past three months**.

24. How often do you typically meet and spend time with friends or family?
Family does **not** include cohabitants such as spouse.
    - ☐ Daily
    - ☐ Several days per week
    - ☐ One or two days per week
    - ☐ One or two days per month
    - ☐ Less often or never

25. How often do you typically communicate via phone, email, text message, chat, or similar means with friends or family?
Family does **not** include cohabitants such as spouse.
    - ☐ Daily
    - ☐ Several days per week
    - ☐ One or two days per week
    - ☐ One or two days per month
    - ☐ Less often or never

26. Do you ever feel bothered by loneliness?
    - ☐ Always
    - ☐ Often
    - ☐ Rarely
    - ☐ Never
    - ☐ Don't know/Prefer not to answer

Social relations and activities

The following questions pertain to how things usually are **on average, over time**.

27. Do you typically do any of the following...

	No	Yes, sometimes	Yes, often
a) Go to the movies, theatre, concerts, museums, exhibitions?	☐	☐	☐
b) Go to restaurants?	☐	☐	☐
c) Help family or friends with baby sitting or other small tasks?	☐	☐	☐
d) Participating in associations, study circles, or courses?	☐	☐	☐
e) Attend religious services or equivalents?	☐	☐	☐
f) Participating in other group activities, group workouts, or singing in a choir?	☐	☐	☐

28. Sometimes, one needs help and support from someone. Do you have any close relative or friend who provide support...
Relative also refers to immediate family members, such as spouse.

	Yes	No
a) …if you fall ill?	☐	☐
b) …if you want company?	☐	☐
c) …if you need to talk to someone about personal concerns?	☐	☐
d) …if you need help with shopping or cooking?	☐	☐

Education and employment

29. What is your highest level of completed formal education?
    - ☐ Elementary, primary, middle school or similar
    - ☐ 2-year high school or higher vocational education
    - ☐ 3–4-year high school
    - ☐ Folk high school or similar
    - ☐ University or college, less than 3 years
    - ☐ University or college, 3 years or longer
    - ☐ Don't know/Prefer not to answer

30. What has been your main occupation or employment during your working career?

____

31. What is your current occupation? Please select all that apply.
    - o Retired
    - o Employed → _ _ _ % of full-time
    - o Self-employed → _ _ _ % of full-time
    - o Other

32. Have you experienced difficulties covering ongoing expenses such as food, rent, bills, etc. in the past 12 months?
    - ☐ No
    - ☐ Yes

Thank you very much for completing the survey!
