# Supplementary material for: The impact of eating alone on food intake and everyday eating routines: A cross-sectional study of community-living 70- to 75-year-olds in Sweden
Source: BMC Public Health. 2024 Aug 14;24:2214. doi: 10.1186/s12889-024-19560-0 (PMC11323640; doi:10.1186/s12889-024-19560-0)
Supplement: Supplementary file 2 — Supplementary Material 2 [file 12889_2024_19560_MOESM2_ESM.pdf]

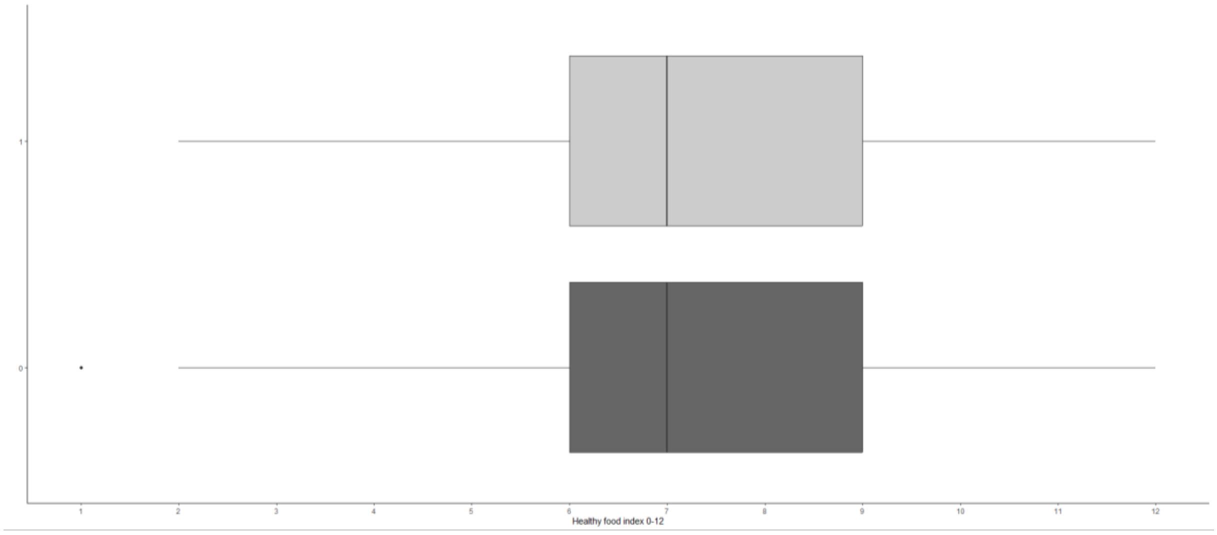

Supplementary Figure 1 Boxplot showing distribution of food index scores (1-12) among those categorised as eating alone (0) or together (1).

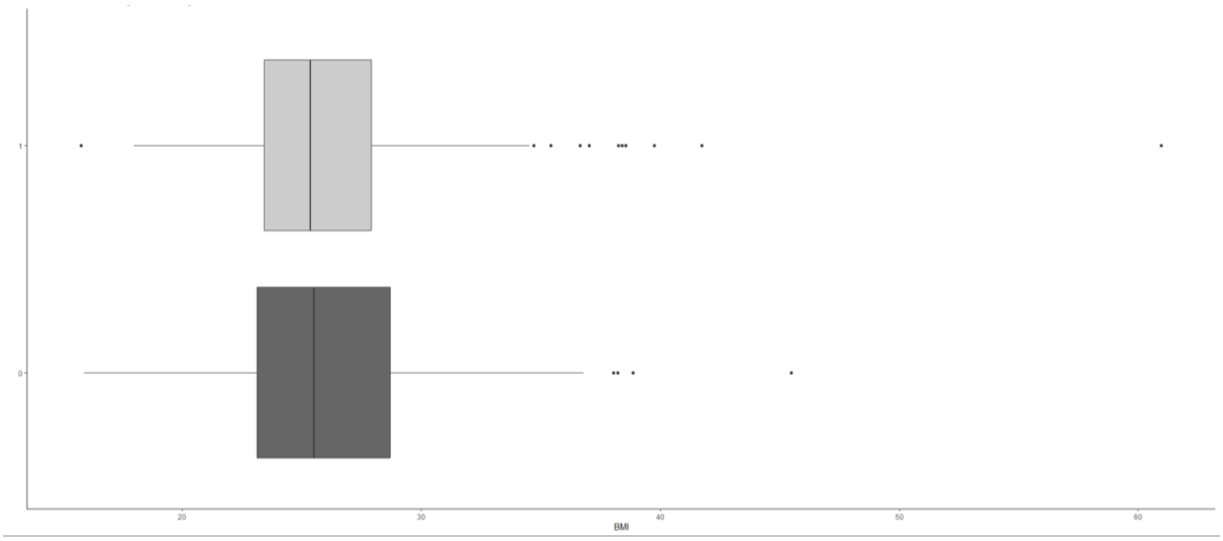

Supplementary Figure 2 Boxplot showing distribution of BMI among those categorised as eating alone (0) or together (1).
